# How are zooplankton’s functional guilds influenced by land use in Amazon streams?

**DOI:** 10.1371/journal.pone.0288385

**Published:** 2023-08-01

**Authors:** Francieli F. Bomfim, Sabrina Deosti, Nayara Louback-Franco, Raimundo L. M. Sousa, Thaisa S. Michelan

**Affiliations:** 1 Laboratório de Ecologia de Produtores Primários, Programa de Pós Graduação em Ecologia, Instituto de Ciências Biológicas, Universidade Federal do Pará, Belém, Pará, Brazil; 2 Laboratório de Zooplâncton, Programa de Pós-Graduação em Ecologia de Ambientes Aquáticos Continentais, Instituto de Ciências Biológicas, Universidade Estadual de Maringá, Maringá, Paraná, Brazil; 3 Programa de Pós-Graduação em Botânica Tropical, Museu Paraense Emílio Goeldi, Universidade Federal Rural da Amazônia, Belém, Pará, Brazil; Central University of South Bihar, INDIA

## Abstract

Amazon streams present great biodiversity and offer several ecosystem services, but these systems are threatened by multiple land uses. The changes created by land use are expected to drive the composition of species, ultimately changing the trophic relationships of several biological groups, including zooplankton. We investigated if land use changes the composition of zooplankton functional guilds in Amazon streams and which are the local (physical-chemical) variables driving the zooplankton functional guilds in the land-use gradient. Zooplankton and physical-chemical variables were sampled in 17 water bodies in the municipality of Barcarena, Pará, Brazil in 2018 and 2019, five sampling sites were in the Pará River and 12 in streams. Forest cover (a proxy for land use) was determined through digital image processing and converted in percentage. Zooplankton species were classified into five functional guilds (filter, raptorial, scraper, suctor, and predator feeders). We recorded 98 zooplankton taxa and filters were the most abundant functional guild. The composition of zooplankton functional guilds did not change in the land use gradient. However, the distribution of zooplankton functional guilds in Amazon streams was determined by local environmental variables related to the feeding strategies. Scraper-feeders (cladocerans) were positively related to greater canopy cover, suctor-feeders and predator-feeders (both rotifers) were related to greater total phosphorus, whereas filter-feeders (rotifers, cladocerans, and copepods) and raptorial (copepods) were related to total suspended solids. This study brings new information about zooplankton in Amazon streams that are under-studied. The functional approach clarifies the patterns observed and reflects the trophic relationships in which the zooplankton community is involved in streams under a degree of land use, i.e., scraper-cladocerans can represent more preserved streams under greater canopy cover, whereas the other functional guilds were related to variables that can represent more altered streams.

## Introduction

Streams are well known for offering several ecosystem services, such as climate regulation, drinking water, food, and recreation [1]. These systems are characterized by hydrologic, geomorphic, and biological processes [2], support great species diversity, and provide species to the regional pool [3, 4]. In some sub-basins of the Amazon territory, streams can represent up to 90% of the total channel length [5], draining large areas inside forests, cities, crops, mining, and others [6–8].

By draining these areas under intense land use, the biodiversity of Amazon streams and their ecosystem services are threatened [9]. That is because stream systems act as a sink catching the changes in the landscape [2, 10]. Deforestation, the input of nutrients, agrochemicals, and mining residuals are examples of threats to the species diversity in Amazon streams [7, 8]. Changes in spatial and local physical-chemical variables of the land and water promoted by land use are followed by changes in the biological groups living in streams, including insects, fish [11], macrophytes [12, 13], and zooplankton [14].

Zooplankton plays many functional roles in freshwater ecosystems [15] and can be used when analyzing the integrity of water bodies because of their fast life cycle and sensitivity to environmental changes [16, 17], such as the ones created by land use. This group participates in the matter and energy flow of different compartments inside water bodies and acts as a link inside the food chains [18, 19]. The three main groups of freshwater mesozooplankton (Copepoda, Cladocera, and Rotifera) explore different sizes and types of food resources depending on their feeding morphology, strategies, feeding preferences, biogeography, and food availability [20–25].

The main representatives of Copepoda in freshwater environments are Calanoids and Cyclopoids. Calanoids are filter feeders preying mainly on algae, whereas Cyclopoids are raptorial/predators preying on other zooplankton organisms [22, 26, 27]. Cladocerans are mainly filters preying on phytoplankton and bacteria, and scraper feeders preying on periphytic organisms and particles [24, 27, 28]. Whereas Rotifera can be predators, suctors, and filter feeders preying on other rotifers, periphytic algae, and phytoplankton, respectively [29–31].

These feeding traits are essential when linking zooplankton to ecosystem functioning as they represent the type of food consumed [20], the feeding behavior (grasping, grinding, pumping, or suction) [32, 33], habitat preferences, metabolic costs linked to predation risks [20], and the role played by these organisms on the elements and energy cycling of different compartments inside aquatic environments [34]. Thus, the functional approach through zooplankton functional guilds can be a straightforward way to analyze how land use is affecting the ecosystem functions, especially thinking about the position that zooplankton occupies inside aquatic food chains linking primary producers and consumers to higher trophic levels [35].

The changes created by land use are expected to drive zooplankton functional guilds in streams. In some environments, the absence of riparian vegetation (due to deforestation) increases the light incidence and nutrient input into the channel, which leads to high primary productivity of algae and macrophytes [12, 36]. This higher productivity favors zooplankton development by offering more food availability [37] and can increase the contribution of filter and raptorial feeders. Whereas preserved streams with great riparian vegetation and low light incidence support less primary productivity, so, the organic material from degrading terrestrial vegetation became more important [38], which can support zooplankton particle feeders such as scrapers and suctors.

Therefore, here, we aimed to answer two questions: 1) Does land use change the composition of zooplankton functional guilds in Amazon streams? For that, we hypothesize that land use would create different environmental conditions on a spatial scale which leads to differences in the composition of zooplankton functional guilds. 2) Which are the local (physical-chemical) variables driving the zooplankton functional guilds in a land-use gradient? For that, we hypothesize that canopy cover and variables related to land use, such as low oxygen concentration, high conductivity, high nutrients concentration, and high temperature would increase filters and predators, whereas more preserved streams with a greater canopy and low temperature would increase scrapers and suctors.

## Methods

### Study area

**Fig 1:**
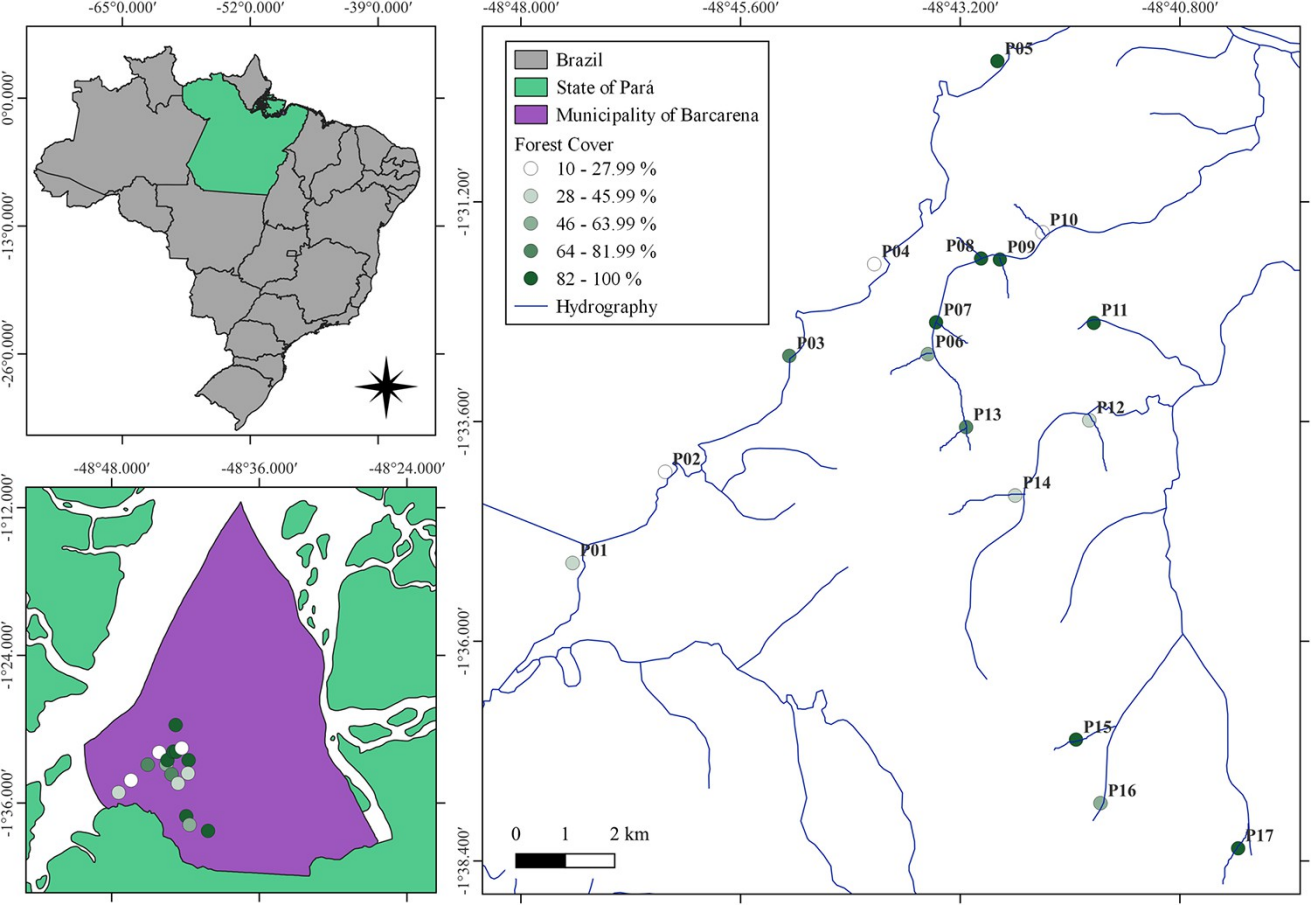
Map of the studied area. Sampling sites with their respective forest cover (%) are shown.

Sampling occurred in the municipality of Barcarena, Pará, Brazil (Fig 1). This region is characterized by a humid tropical climate with rainfall during the entire year. The average annual rainfall is between 2,300 to 2,800 mm and the average temperature is 27°C [39]. Barcarena is inside the world’s largest remaining tropical forest, the Amazon, but its aquatic environments are threatened by multiple land uses, such as agriculture and industrial activities [40]. This municipality became an important economic center for the Pará State since the implementation of industries for ores processing and fertilizers production, among others [41]. The industrial expansion led to an increase in the port areas to easily transport these products, which also led to changes in the aquatic environments connected to those ports, such as the Pará River [40, 41]. The sites analyzed were inside urbanized and industrialized areas as well as primary and secondary forests that are close to agriculture and pasture fields. We did not need formal authorization to access the field sites as they are inside public areas or private properties that we had a previous agreement with the owner.

### Zooplankton sampling and laboratory analysis

Zooplankton and water were sampled in 17 water bodies, five sampling sites were in the Pará River and twelve were in streams, each sampling site was at least 1 km apart from the other (Fig 1). The first sampling was in November 2018, the second was in February 2019, and the third was in March 2019.

Zooplankton samples were taken at the subsurface (approximately 30 cm below the water-air interface), in the central region of the water bodies. At each sampled site, 100 liters of water were filtered through a plankton net (68 μm) with buckets and fixed with 4% formalin solution buffered with calcium carbonate [42, 43]. Specialized bibliographies were used for species identification [44–46]. To determine the zooplankton abundance (individuals/m^3^), we used an optical microscope (Olympus CX-41) and a Sedgewick-Rafter camera. At least 50 individuals from each zooplankton group (rotifers, cladocerans, and copepods) were counted in each sample. When the samples did not reach this minimum number of individuals, they were fully counted. Zooplankton organisms were classified into five functional groups related to their feeding strategies [30, 47]: filters (that included cladocerans, copepods calanoid, and rotifers), scrapers (cladocerans), suctors (rotifers), raptorial (copepods cyclopoid), and predator-rotifer (Table 1).

**Table 1:** Functional guilds. Description of each functional guild with their main food preferences and strategies.

Functional guild	Zooplanktonic group	Description of food preferences and feeding strategies
**Filter**	Rotifera	Group of planktonic organisms that feed mostly on suspended algae. These organisms create movements in the water by moving their crown of cilia and then the particles are collected by the mouth apparatus (mastax) and filtered [29, 33].
	Copepoda Calanoida	Group of planktonic organisms that feed when the water current is passing through their filtering structure (filter). They feed on algae, bacteria, and small particles [16, 48].
	Cladocera	Group of planktonic cladocerans that can feed on algae, bacteria, and suspended material. Feed with an apparatus on the thoracic appendices or legs [16, 27].
**Scraper**	Cladocera	This group feeds by scraping algal particles from surfaces, it is favored by macrophytes and other substrates [27].
**Suctor**	Rotifera	These rotifers project their mouthparts (mastax) outwards and thus pierce the algae to feed, they live more associated with substrates and macrophytes [29].
**Raptorial**	Copepoda	This group’s prey (such as protozoa) is actively captured and killed [27]. They have buccal appendages with adaptations for capturing prey [49] and have a preference for larger particles [16].
	Cyclopoida	
**Predator**	Rotifera	This group’s main diet consists of other rotifers, cladocerans, copepods, and ciliates [50, 51]. They feed by processing the food with receptors on their corona, they use the mastax to ingest the food and then stuff the particles into their stomach with the trophi [33, 51].

### Environmental characterization

Water physical-chemical parameters were measured using a multiparameter probe (Horiba U-50) and consisted of pH, water temperature (°C), conductivity (μS/cm), and dissolved oxygen (mg/L). Additionally, we collected 1L water samples in the water column (approximately 30 cm) and froze them for laboratory analysis. We used the water sample to determine the alkalinity, BOD (Biochemical demand of oxygen, 20°C mg/L, i.e., oxygen consumed by microorganisms while they decompose organic matter under aerobic conditions), total phosphorus (mg/L), nitrate (NO_3-_ mg/L), ammonia (NH_3_mg/L), and total suspended solids (mg/L) following the protocol from the American Water and Waste Association’s Standard Method for the Examination of Water and Waste-Water [52]. We also measured the canopy cover above the sampling points using a densitometer, which we later converted to percentages [53]. The average of each environmental variable analyzed is shown in the S1 Table.

Forest cover was calculated from the mosaic images of the RapidEye Earth Imaging System (REIS) optical sensor obtained for the sampling period (2018–2019). We performed Digital Image Processing to classify the percentage of forest cover (primary and secondary forest) in the PCI Geomatics software, using the ATCOR Ground Reflectance module [54]. After atmospheric image correction, the REIS mosaic was generated in the OrtoEngine module of PCI Geomatics. The data were validated by comparison with TerraClass 2014 images provided by the National Institute for Space Research (INPE) [55] and in situ observation. The forest cover for each sampling site was obtained from a 150 m wide linear buffer (total width 300 m) with a length of 300 m upstream and 300 m downstream from a central geographical coordinate determined at each point.

### Statistical analysis

To answer our first question (does land use change the composition of zooplankton functional guilds in Amazon streams?) we performed a PERMANOVA (Permutational analysis of variance) using the abundance of each functional group as the response variable and the percentage of forest cover (a proxy for land use) as the predictor. Abundances were log-transformed before applying the Bray-Curtis distance, we used the ‘adonis2’ function from the “vegan” package [56] and applied 999 permutations.

To answer our second question (which are the physical-chemical variables driving the zooplankton functional guilds in a land-use gradient?) we performed a Redundancy analysis (RDA). Before running the RDA, the matrix with the abundance of functional guilds (response variable) was transformed into a Hellinger relative abundance matrix. We tested multicollinearity among environmental variables using VIF (variance inflation factor) with the function ‘vif.cca’ from the “vegan” package in R. The variables presented no correlation (VIF<10). The final set of environmental variables was selected through the forward selection method with two stopping rules [57] using the ‘ordistep’ function from the “vegan” package. This analysis avoids overestimating residuals in canonical analyzes (such as Redundancy Analysis) generated by non-explanatory variables [57]. From all environmental variables, only canopy cover, total phosphorus, total suspended solids, alkalinity, pH, NO_3-_, oxygen dissolved, and temperature were selected as the predictors in the RDA analysis. The RDA model, the axes, and each term of the model were tested by an ANOVA using the functions ‘anova’ and ‘anova.cca’ from “vegan”. All graphics were performed using the “ggplot2” package [58] and the ‘ggplot’ function. All analyses were performed in R version 4.0.2 and R Studio version 1.3.1093 [59]. The significance for all analyses was p<0.05.

## Results

We recorded 98 zooplankton taxa, rotifers were the most representative with 53 taxa, followed by cladocerans with 30, and copepods with 15. The species list with the classification into the functional guilds is shown in the S2 Table. Filter-rotifer was the most abundant guild (42.65%), followed by filter-Cladocera (32.44%), suctor (9.25%), scraper (8.42%), raptorial (7.29%), filter-Copepoda (0.86%), and predator-rotifer (0.07%). The composition of zooplankton functional guilds did not differ in the gradient of forest cover (a proxy for land use) (p = 0.07; Table 2), i.e., the functional guilds were not influenced by the spatial variable.

**Table 2:** PERMANOVA results. The effect of forest cover on the composition of zooplankton functional guilds. Df: degrees of freedom.

	Df	R^2^	F-value	p-value
Forest cover (%)	1	0.04	2.15	0.071
Residual	49	0.96		
Total	50	1		

**Fig 2:**
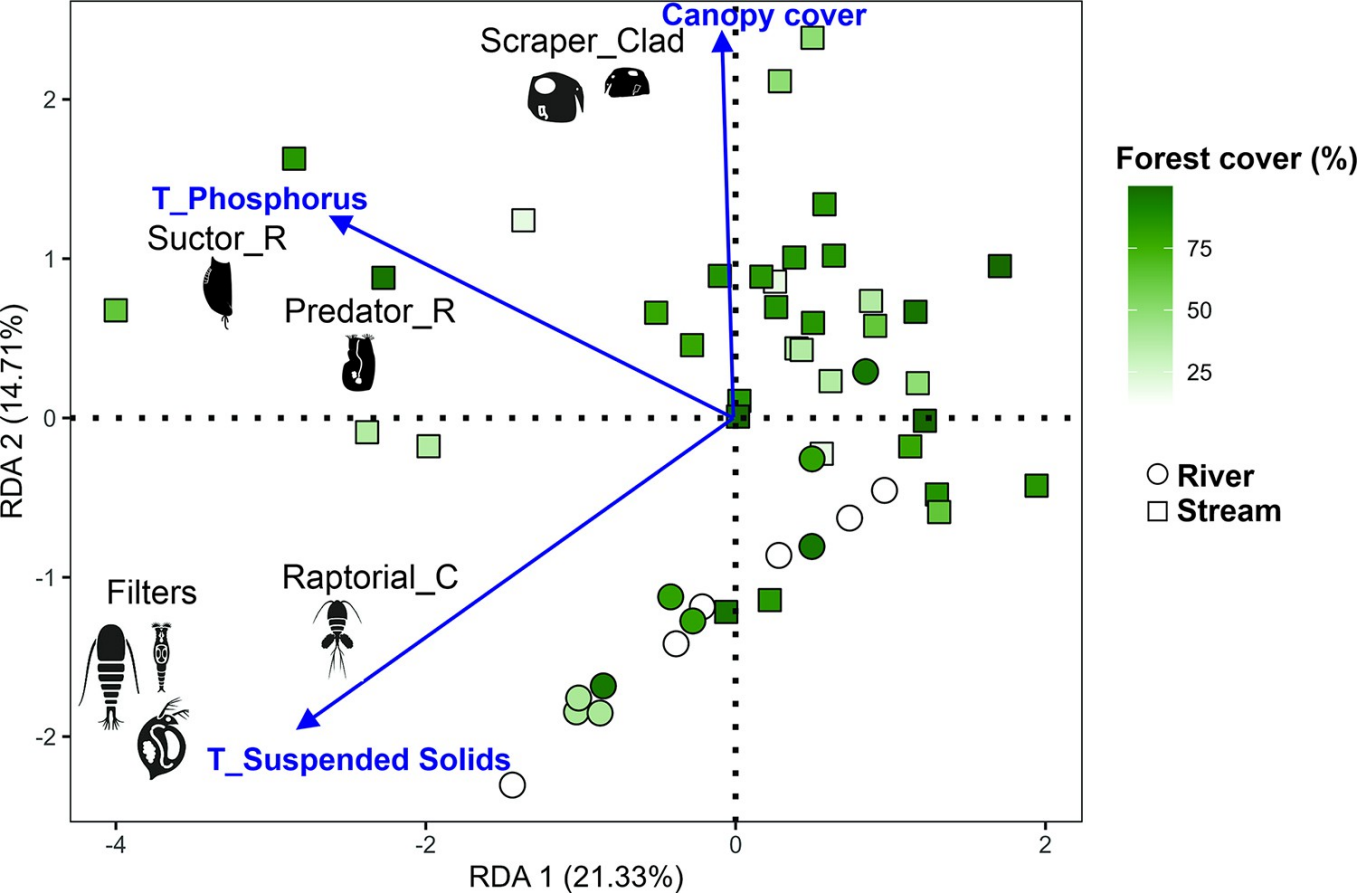
RDA representation with the environmental variables and zooplankton functional groups in the gradient of land use (forest cover). Scraper_Clad = Scraper-Cladocera, Suctor_R = Suctor-Rotifera, Predator_R = Predator-Rotifera, Raptorial_Cop = Raptorial-Copepoda, T_Phosphorus = Total phosphorus, T_Suspended Solids = Total suspended solids. Only significant relationships are shown.

The environmental variables in the RDA model explained 36% of the variation in the zooplankton functional guilds in the land-use gradient (global model: F = 2.933, p = 0.001). The first axis retained 21.33% of the explanation (p<0.001) and the second axis 14.71% of the explanation (p<0.001). Only three environmental variables significantly influenced the functional guilds: canopy cover, total phosphorus, and total suspended solids (Table 3). Scraper-feeders (cladocerans) were positively related to greater canopy cover, suctor-feeders and predator-feeders (both rotifers) were related to greater total phosphorus, whereas filter-feeders (rotifers, cladocerans, and copepods) and raptorial (copepods) were related to total suspended solids (Fig 2).

**Table 3:** RDA results. Output from ANOVA testing each term inside the RDA model. Df: degrees of freedom. Bold represents significant values (p< 0.05).

Environmental Variables	Df	F-value	p-value
Canopy cover (%)	1	6.91	**0.001**
Total Phosphorus (mg/L)	1	5.58	**0.002**
Total Suspended Solids (mg/L)	1	6.45	**0.001**
Alkalinity	1	1.75	0.147
pH	1	1.37	0.264
NO_-3_ (mg/L)	1	0.82	0.516
Dissolved oxygen (mg/L)	1	1.59	0.163
Temperature (°C)	1	0.46	0.765

## Discussion

Land use can transform landscapes and alter species composition, ultimately changing the trophic structure of freshwater environments [38, 60], however, here, the forest cover (a proxy for land use) did not change the composition of zooplankton functional guilds, as expected. On the other hand, the distribution of zooplankton functional guilds in Amazon streams was determined by local environmental variables related to the feeding strategies (canopy cover, suspended solids, and phosphorus), corroborating our second hypothesis.

Spatial variables can be important for zooplankton composition [61] and are especially related to environmental heterogeneity and dispersion [62, 63]. Greater spatial extents are usually related to greater environmental heterogeneity creating different conditions related to food availability and water physical-chemical variables [62, 64], which can select a different set of species and lead to differences in zooplankton composition [65]. A great spatial extent can also impose limitations on species dispersion if the water bodies are not connected [63]. Our spatial variable–forest cover–did not change the composition of zooplankton functional groups. We believe that the spatial extent studied was not great enough to catch extremes in environmental heterogeneity and impose limitations on zooplankton dispersion. So, the absent relationship between zooplankton composition and forest cover could be a reflex of stochastic factors acting (such as birth, mortality, colonization, and extinction [66]). Furthermore, zooplankton organisms present diverse trophic and spatial niches that make them more resilient to anthropogenic pressures and can favor them in more degraded environments [67], which could lead to a constant composition of functional guilds on a spatial scale. Gomes et al. [14] analyzed zooplankton composition in the south Amazon in streams inserted in a gradient of forest cover and also did not find a relationship between spatial variables and the zooplankton community, reaffirming the need to further investigate other variables besides forest cover.

Regarding the local variables analyzed, canopy cover is an important variable to structure the productivity of streams and change trophic relationships [36, 38]. Greater canopy openness allows high light incidence in the channel and the establishment of algae and macrophytes that offer food resources to other communities, whereas the food webs under greater canopy closeness are more dependent on allochthonous organic material present on the substrate [38, 68]. We observed a similar pattern with scrapers-cladocerans positively related to canopy cover, these organisms feed on organic particles, especially periphyton algae present in substrates [27], and can be an important part of the zooplankton community in freshwater environments. Also, small-bodied cladocerans such as scraper feeders can substitute algae for organic particles (1–15 μm) as a food source, when algal production is low, giving them an advantage in this scenario [28]. Some studies with macroinvertebrates’ trophic structure have also reported similar findings, where there is a predominance of organisms that feed on small organic particulate matter in streams under greater riparian forest and canopy cover [38, 60, 69, 70].

Phosphorus concentration was positively related to rotifers’ suctor and predator, this variable indicates a high potential for productivity as together with nitrogen and potassium are the key elements to promote the development of algae and macrophytes in freshwater environments [71, 72]. High algae and macrophytes productivity can also allow high secondary productivity by offering more food resources [72, 73], which could explain the relationship between phosphorus and rotifer predators that feed on other rotifers and suctor-rotifers that are usually associated with macrophytes and feed on algae, as they would be able to explore different food resources with this association [74]. Phosphorus is also an essential element for the metabolism of zooplankton species and rotifers are known for maintaining high tolerance in eutrophic environments [31] i.e., at high phosphorus concentrations.

The other variable selected by the RDA, suspended solids, has a close relationship with zooplankton feeding as it can disrupt the filtration apparatus of filter feeders [28] or represent higher availability of food items, as suspended solids can include bacteria, algae, and other organic particles [75]. The food availability explains the positive relationship observed between the filter and raptorial feeders and suspended solids in the streams studied. High concentrations of suspended solids are usually observed in more altered environments with a great input of nutrients and floating particles, some of them are characterized as eutrophic [76]. Each group of zooplankton filter feeders has a different strategy, rotifers can be more food selective than cladocerans and can avoid the ingestion of clay particles, so they are not inhibited by suspended clay and are usually in overwhelming dominance [28]. Copepods, both filter and raptorial are very selective [27, 77] and have already been related to suspended solids in other studies [25, 76]. Another point to be considered is that high concentrations of suspended solids can turn the water heavily colored allowing zooplankton to escape the predation of small-bodied fish that are visual hunters [78, 79], reaffirming the positive relationship observed.

Rotifer filter feeders were the most abundant group and the richest in species number. Rotifers are well-known as r-strategists and can present fast growth even at low food concentrations giving them an advantage over cladocerans and copepods that need more time to develop [16, 29]. Also, this group can feed on detritus that they select, and in the absence of zooplankton competitors and predators, they can establish an abundant and rich community [80, 81]. Cladocerans and copepods are the opposite, they have slower development and because of their large body sizes are more predated by small fish [82] present in streams, which could reflect in their lower abundance in the functional guilds compared to rotifers.

This study brings new information about the occurrence and distribution of zooplankton in Amazon streams and helps to better understand how zooplankton functional guilds can be related to local environmental variables in streams under different degrees of land use. The functional approach clarifies the patterns observed and reflects the trophic relationships in which the zooplankton community is involved. Thus, scraper-cladocerans can represent more preserved streams under greater canopy cover, whereas the other functional guilds were related to variables that can represent more altered streams. Besides ecological interactions, the distribution of different zooplankton functional guilds is also related to the energy and matter cycling on different water body compartments [35]. Cladocerans scrapers and rotifer suctors, for example, are more related to the littoral zone and interact with macrophytes, periphytic algae, and macroinvertebrates [27, 32]. Whereas raptorial copepods, rotifer predators, and filters are more related to the pelagic zone, feeding on phytoplankton and developing strategies to escape predation by small fishes that are visual hunters [33, 34, 83]. Future studies including other variables related to land use and food availability can better clarify the observed patterns and help to understand the functional role that zooplankton is playing in streams under anthropogenic pressure, especially in Amazon streams that are a system under-studied.

## Supporting information

S1 Table Environmental variables analyzed. The average ± standard deviation is shown for each variable measured. pH, alkalinity, BOD = (Biochemical demand of oxygen, 20°C mg/L), T_P = total phosphorus (mg/L), NO3 = nitrate (NO_3-_ mg/L), NH3 = ammonia (NH_3_mg/L), T_SS = total suspended solids (mg/L), temperature (°C), conductivity (μS/cm), DO = dissolved oxygen (mg/L), and canopy cover (%).
(DOCX)

S2 Table List of taxa. The taxa recorded are classified into their respective functional guild.
(DOCX)
