# Supplementary material for: How are zooplankton’s functional guilds influenced by land use in Amazon streams?
Source: PLoS One. 2023 Aug 1;18(8):e0288385. doi: 10.1371/journal.pone.0288385 (PMC10393134; doi:10.1371/journal.pone.0288385)
Supplement: S1 Table — The average ± standard deviation is shown for each variable measured. pH, alkalinity, BOD = (Biochemical demand of oxygen, 20°C mg/L), T_P = total phosphorus (mg/L), NO3 = nitrate (NO3- mg/L), NH3 = ammonia (NH3mg/L), T_SS = total suspended solids (mg/L), temperature (°C), conductivity (μS/cm), DO = dissolved oxygen (mg/L), and canopy cover (%). (DOCX) [file pone.0288385.s001.docx]

Supporting information

**Table S1. Environmental variables analyzed.** The average ± standard deviation is shown for each variable measured. pH, alkalinity, BOD= (Biochemical demand of oxygen, 20 °C mg/L), T_P= total phosphorus (mg/L), NO3= nitrate (NO_3-_ mg/L), NH3= ammonia (NH_3_mg/L), T_SS= total suspended solids (mg/L), temperature (ºC), conductivity (µS/cm), DO= dissolved oxygen (mg/L), and canopy cover (%).

| **Sites** | **pH** | **Alkalinity** | **BOD** | **T_P** | **NO3** | **NH3** | **T_SS** | **Temperature** | **Conductivity** | **DO** | **Canopy cover** |
| --- | --- | --- | --- | --- | --- | --- | --- | --- | --- | --- | --- |
| **P01** | 7.00 ± 0.17 | 14.67 ± 4.16 | 54.90 ± 40.87 | 0.00 ± 0.00 | 0.47 ± 0.25 | 0.30 ± 0.00 | 13.67 ± 6.51 | 26.97 ± 0.25 | 0.03 ± 0.06 | 7.20 ± 0.70 | 0.00 ± 0.00 |
| **P02** | 7.63 ± 0.49 | 16.00 ± 3.46 | 44.60 ± 19.34 | 0.10 ± 0.17 | 0.37 ± 0.25 | 0.43 ± 0.06 | 6.67 ± 2.08 | 31.03 ± 2.45 | 0.10 ± 0.00 | 6.40 ± 0.90 | 0.00 ± 0.00 |
| **P03** | 8.20 ± 0.23 | 18.00 ± 3.46 | 52.20 ± 38.66 | 0.00 ± 0.00 | 0.40 ± 0.10 | 0.27 ± 0.12 | 31.67 ± 21.01 | 27.80 ± 1.20 | 0.10 ± 0.00 | 6.33 ± 0.25 | 0.00 ± 0.00 |
| **P04** | 7.44 ± 0.15 | 14.00 ± 2.00 | 46.90 ± 46.38 | 0.00 ± 0.00 | 0.43 ± 0.06 | 0.17 ± 0.06 | 5.00 ± 1.00 | 30.20 ± 1.20 | 0.07 ± 0.06 | 6.73 ± 0.45 | 0.00 ± 0.00 |
| **P05** | 6.87 ± 0.03 | 14.00 ± 3.46 | 49.87 ± 42.20 | 0.00 ± 0.00 | 0.37 ± 0.06 | 0.27 ± 0.12 | 18.33 ± 16.44 | 27.50 ± 0.50 | 0.00 ± 0.00 | 6.30 ± 0.20 | 0.00 ± 0.00 |
| **P06** | 5.57 ± 0.00 | 18.00 ± 12.49 | 35.50 ± 29.70 | 0.20 ± 0.35 | 0.33 ± 0.06 | 0.27 ± 0.12 | 6.67 ± 4.51 | 26.00 ± 0.00 | 0.20 ± 0.00 | 2.60 ± 0.00 | 60.33 ± 52.26 |
| **P07** | 5.54 ± 0.39 | 13.33 ± 7.57 | 34.90 ± 15.69 | 0.07 ± 0.12 | 0.67 ± 0.06 | 0.37 ± 0.15 | 9.00 ± 8.66 | 25.60 ± 0.60 | 0.13 ± 0.06 | 3.47 ± 0.15 | 60.47 ± 52.41 |
| **P08** | 5.33 ± 0.35 | 9.33 ± 1.15 | 40.60 ± 30.09 | 0.47 ± 0.55 | 0.40 ± 0.10 | 0.50 ± 0.35 | 24.67 ± 33.50 | 25.30 ± 0.30 | 0.00 ± 0.00 | 6.00 ± 1.50 | 92.17 ± 2.95 |
| **P09** | 5.62 ± 0.29 | 6.00 ± 2.00 | 49.73 ± 52.52 | 0.57 ± 0.72 | 1.30 ± 0.22 | 1.00 ± 1.47 | 7.67 ± 3.79 | 25.10 ± 0.70 | 0.00 ± 0.00 | 6.53 ± 1.85 | 56.30 ± 7.37 |
| **P10** | 6.09 ± 0.14 | 10.00 ± 6.93 | 31.27 ± 23.70 | 0.40 ± 0.52 | 1.70 ± 0.04 | 1.27 ± 1.42 | 13.33 ± 15.31 | 26.57 ± 2.15 | 0.07 ± 0.06 | 4.87 ± 1.15 | 9.83 ± 2.66 |
| **P11** | 5.39 ± 0.00 | 5.00 ± 3.00 | 52.00 ± 47.70 | 0.13 ± 0.06 | 0.50 ± 0.20 | 0.23 ± 0.15 | 2.00 ± 0.00 | 27.20 ± 0.00 | 0.00 ± 0.00 | 8.60 ± 0.00 | 57.63 ± 49.93 |
| **P12** | 6.78 ± 0.01 | 12.00 ± 2.00 | 52.33 ± 41.48 | 0.37 ± 0.46 | 0.60 ± 0.46 | 0.57 ± 0.72 | 1.67 ± 0.58 | 29.43 ± 0.06 | 1.90 ± 0.90 | 6.17 ± 0.65 | 91.33 ± 3.50 |
| **P13** | 5.21 ± 0.41 | 6.00 ± 2.00 | 31.63 ± 33.95 | 0.23 ± 0.06 | 0.90 ± 0.36 | 0.50 ± 0.17 | 1.00 ± 1.00 | 25.07 ± 0.75 | 0.03 ± 0.06 | 4.53 ± 2.25 | 65.57 ± 11.27 |
| **P14** | 5.45 ± 0.22 | 7.33 ± 7.57 | 41.27 ± 37.04 | 0.97 ± 0.21 | 0.80 ± 0.61 | 0.10 ± 0.10 | 18.33 ± 22.37 | 24.90 ± 0.70 | 0.00 ± 0.00 | 5.87 ± 1.75 | 72.10 ± 6.41 |
| **P15** | 5.30 ± 0.02 | 10.67 ± 9.87 | 52.77 ± 42.01 | 0.00 ± 0.00 | 0.40 ± 0.26 | 0.50 ± 0.10 | 1.67 ± 0.58 | 24.73 ± 0.15 | 0.00 ± 0.00 | 6.13 ± 1.75 | 89.63 ± 2.75 |
| **P16** | 4.94 ± 0.28 | 6.00 ± 5.29 | 23.47 ± 26.46 | 0.47 ± 0.64 | 0.93 ± 0.49 | 0.33 ± 0.49 | 2.33 ± 1.15 | 26.00 ± 0.20 | 0.00 ± 0.00 | 5.83 ± 2.45 | 92.40 ± 2.99 |
| **P17** | 5.16 ± 0.22 | 7.33 ± 6.11 | 27.07 ± 32.46 | 0.83 ± 1.01 | 1.00 ± 0.95 | 0.87 ± 1.07 | 3.00 ± 4.36 | 24.63 ± 0.85 | 0.00 ± 0.00 | 6.67 ± 0.95 | 86.57 ± 0.58 |
