# Supplementary material for: How are zooplankton’s functional guilds influenced by land use in Amazon streams?
Source: PLoS One. 2023 Aug 1;18(8):e0288385. doi: 10.1371/journal.pone.0288385 (PMC10393134; doi:10.1371/journal.pone.0288385)
Supplement: S2 Table — The taxa recorded are classified into their respective functional guild. (DOCX) [file pone.0288385.s002.docx]

Supporting information

**Table S2. List of taxa.** The taxa recorded are classified into their respective functional guild.

| **Taxa list** | **Functional guild** |
| --- | --- |
| *Asplanchna sieboldi (Leydig, 1854)* | Predator-R |
| *Brachionus caudatus Barrois & Daday, 1894* | Filtration-R |
| *Brachionus mirus Daday, 1905* | Filtration-R |
| *Brachionus quadridentatus Hermann, 1783* | Filtration-R |
| *Brachionus zahniseri Ahlstrom, 1934* | Filtration-R |
| *Brachionus falcatus Zacharias, 1898* | Filtration-R |
| *Brachionus calyciflorus Pallas, 1766* | Filtration-R |
| *Brachionus urceolaris Müller, 1773* | Filtration-R |
| *Keratella cochlearis (Gosse, 1851)* | Filtration-R |
| *Keratella americana Carlin, 1943* | Filtration-R |
| *Plationus patulus (Müller, 1786)* | Filtration-R |
| *Platyias quadricornis (Ehrenberg, 1832)* | Filtration-R |
| *Platyias leloupi Gillard, 1957* | Filtration-R |
| *Beauchampiella eudactylota (Gosse, 1886)* | Filtration-R |
| *Dipleuchlanis propatula (Gosse, 1886)* | Filtration-R |
| *Euchlanis incisa Carlin, 1939* | Filtration-R |
| *Euchlanis dilatata Ehrenberg, 1832* | Filtration-R |
| *Filinia longiseta (Ehrenberg, 1834)* | Filtration-R |
| *Filinia opoliensis (Zacharias, 1898)* | Filtration-R |
| *Filinia terminalis (Plate, 1886)* | Filtration-R |
| *Lecane furcata (Murray, 1913)* | Filtration-R |
| *Lecane bulla (Gosse, 1851)* | Filtration-R |
| *Lecane cornuta (Müller, 1786)* | Filtration-R |
| *Lecane curvicornis (Murray, 1913)* | Filtration-R |
| *Lecane leontina (Turner, 1892)* | Filtration-R |
| *Lecane elsa Hauer, 1931* | Filtration-R |
| *Lecane lunaris (Ehrenberg, 1832)* | Filtration-R |
| *Lecane proiecta Hauer, 1956* | Filtration-R |
| *Lecane papuana (Murray, 1913)* | Filtration-R |
| *Lecane quadridentata (Ehrenberg, 1830)* | Filtration-R |
| *Lecane hornemanni (Ehrenberg, 1834)* | Filtration-R |
| *Lecane amazonica (Murray, 1913)* | Filtration-R |
| *Lecane luna (Müller, 1776)* | Filtration-R |
| *Lepadella ovalis (Müller, 1786)* | Filtration-R |
| *Mytilina macrocera (Jennings, 1894)* | Filtration-R |
| *Enteroplea lacustris Ehrenberg, 1830* | Sugador-R |
| *Notommata falcinella Harring & Myers, 1922* | Sugador-R |
| *Notommata copeus Ehrenberg, 1834* | Sugador-R |
| *Notommata pachyura (Gosse, 1886)* | Sugador-R |
| *Notommata cerberus (Gosse, 1886)* | Sugador-R |
| *Notommata sp* | Sugador-R |
| *Scaridium grandis Segers, 1995* | Predator-R |
| *Polyarthra dolichoptera Idelson, 1925* | Filtration-R |
| *Hexarthra mira (Hudson, 1871)* | Filtration-R |
| *Synchaeta pectinata Ehrenberg, 1832* | Filtration-R |
| *Testudinella patina (Hermann, 1783)* | Filtration-R |
| *Trichocerca chattoni (de Beauchamp, 1907)* | Sugador-R |
| *Trichocerca gracilis (Tessin, 1890)* | Sugador-R |
| *Trichocerca similis (Wierzejski, 1893)* | Sugador-R |
| *Trichocerca dixonnutalli (Jennings, 1903)* | Sugador-R |
| *Trichotria tetractis (Ehrenberg, 1830)* | Sugador-R |
| *Macrochaetus collinsii (Gosse, 1867)* | Filtration-R |
| *Bdelloidea* | Filtration-R |
| *Bosmina hagmanni Stingelin, 1904* | Filtration-Clad |
| *Bosminopsis deitersi Richard, 1895* | Filtration-Clad |
| *Alona guttata Sars, 1862* | Scraper-Clad |
| *Biapertura ossiani (Sinev, 1998)* | Scraper-Clad |
| *Ovalona glabra Sars, 1901* | Scraper-Clad |
| *Alonella dadayi Birge, 1910* | Scraper-Clad |
| *Anthalona verrucosa (G.O.Sars, 1901)* | Scraper-Clad |
| *Anthalona sp* | Scraper-Clad |
| *Chydorus eurynotus Sars, 1901* | Scraper-Clad |
| *Chydorus nitidulus (Sars, 1901)* | Scraper-Clad |
| *Chydorus pubescens Sars, 1901* | Scraper-Clad |
| *Chydorus parvireticulatus Frey, 1987* | Scraper-Clad |
| *Chydorus sp* | Scraper-Clad |
| *Coronatella monacantha Sars, 1901* | Scraper-Clad |
| *Coronatella poppei Richard, 1897* | Scraper-Clad |
| *Euryalona brasiliensis Brehm & Thomsen, 1936* | Scraper-Clad |
| *Nicsmirnovius paggii Sousa & Elmoor-Loureiro, 2017* | Scraper-Clad |
| *Notoalona sculpta (Sars, 1901)* | Scraper-Clad |
| *Oxyurella ciliata Bergamin, 1931* | Scraper-Clad |
| *Ephemeroporus hybridus (Daday, 1905)* | Scraper-Clad |
| *Ephemeroporus barroisi (Richard, 1894)* | Scraper-Clad |
| *Ceriodaphnia cornuta Sars, 1885* | Filtration-Clad |
| *Diaphanosoma spinulosum Herbst, 1975* | Filtration-Clad |
| *Diaphanosoma brevireme Sars, 1901* | Filtration-Clad |
| *Ilyocryptus spinifer Herrick, 1882* | Filtration-Clad |
| *Macrothrix laticornis (Jurine, 1820)* | Scraper-Clad |
| *Macrothrix sp* | Scraper-Clad |
| *Macrothrix squamosa Sars, 1901* | Scraper-Clad |
| *Moina minuta Hansen, 1899* | Filtration-Clad |
| *Sarsilatona behningi Korovchinsky, 1985* | Filtration-Clad |
| *Cyclopoida sp* | Raptorial-Cop |
| *Ectocyclops herbsti Dussart, 1984* | Raptorial-Cop |
| *Eucyclops elegans (Herrick, 1884)* | Raptorial-Cop |
| *Mesocyclops ogunnus Onabamiro, 1957* | Raptorial-Cop |
| *Mesocyclops sp* | Raptorial-Cop |
| *Paracyclops chiltoni (Thomson G.M., 1883)* | Raptorial-Cop |
| *Tropocyclops prasinus (Fischer, 1860)* | Raptorial-Cop |
| *Tropocyclops sp* | Raptorial-Cop |
| *Microcyclops finitimus Dussart, 1984* | Raptorial-Cop |
| *Thermocyclops decipiens (Kiefer, 1929)* | Raptorial-Cop |
| *Notodiaptomus amazonicus (Wright, 1935)* | Filtration-Cop |
| *Notodiaptomus henseni (Dahl F., 1894)* | Filtration-Cop |
| *Diaptomus frutosae Perbiche-Neves & Boxshall, 2013* | Filtration-Cop |
| *Pseudodiaptomus sp* | Filtration-Cop |
| *Calanoida sp* | Filtration-Cop |
